# Local hemostasis, immunothrombosis, and systemic disseminated intravascular coagulation in trauma and traumatic shock

**DOI:** 10.1186/s13054-015-0735-x

**Published:** 2015-02-23

**Authors:** Satoshi Gando, Yasuhiro Otomo

**Affiliations:** Division of Acute and Critical Care Medicine, Department of Anesthesiology and Critical Care Medicine, Hokkaido University Graduate School of Medicine, N15W7, Kitaku, Sapporo, 060-8638 Japan; Department of Acute Critical Care and Disaster Medicine, Tokyo Medical and Dental University, Yushima 1-5-45, Bunkyoku, Tokyo 113-8510 Japan

## Abstract

Knowing the pathophysiology of trauma-induced coagulopathy is important for the management of severely injured trauma patients. The aims of this review are to provide a summary of the recent advances in our understanding of thrombosis and hemostasis following trauma and to discuss the pathogenesis of disseminated intravascular coagulation (DIC) at an early stage of trauma. Local hemostasis and thrombosis respectively act to induce physiological wound healing of injuries and innate immune responses to damaged-self following trauma. However, if overwhelmed by systemic inflammation caused by extensive tissue damage and tissue hypoperfusion, both of these processes foster systemic DIC associated with pathological fibrin(ogen)olysis. This is called DIC with the fibrinolytic phenotype, which is characterized by the activation of coagulation, consumption coagulopathy, insufficient control of coagulation, and increased fibrin(ogen)olysis. Irrespective of microvascular thrombosis, the condition shows systemic thrombin generation as well as its activation in the circulation and extensive damage to the microvasculature endothelium. DIC with the fibrinolytic phenotype gives rise to oozing-type non-surgical bleeding and greatly affects the prognosis of trauma patients. The coexistences of hypothermia, acidosis, and dilution aggravate DIC and lead to so-called trauma-induced coagulopathy.

*He that would know what shall be must consider what has been.*

*The Analects of Confucius.*

## Introduction

A half century ago, the concept of disseminated intravascular coagulation (DIC) was ridiculed to be an abbreviation for ‘disseminated international confusion’ because intravascular thrombosis was hardly ever found at autopsy. At the end of the 1970s, however, Spero and colleagues [1] correctly pronounced that DIC equals a sign that ‘death is coming’. Since then, DIC has been recognized as an independent disease entity caused by diverse insults, including trauma and sepsis [2]. Recent findings suggest that, under certain circumstances, local thrombosis as well as hemostasis is a physiological process that constitutes an intrinsic effector mechanism of innate immunity, which is called immunothrombosis [3]. DIC is now believed to be a result of the dysregulation of immunothrombosis. This suggests that all insults, irrespective of whether they are infectious (sepsis) or non-infectious (trauma), can cause DIC when the control mechanisms of immunothrombosis are overwhelmed.

However, the new theory of acute coagulopathy of trauma shock (ACOTS) has completely denied DIC, indicating that there is nothing to suggest a process of DIC in the development of ACOTS and that the DIC terminology to trauma is unhelpful and counterproductive [4,5]. Naturally, rebuttals to this concept have been published [6]. The Scientific and Standardization Committee (SSC) on DIC of the International Society on Thrombosis and Haemostasis (ISTH) has published one concept and six considerations for discussion about the hemostatic changes in trauma [7]. After this announcement, the SSC on DIC of the ISTH concluded that the available data suggest that ACOTS is not a new concept but a disease entity similar to or the same as DIC with the fibrinolytic phenotype at an early stage of trauma [8]. A principal component analysis of coagulation after trauma demonstrated that consumption coagulopathy, namely DIC, predicts mortality, the prothrombin time international normalized ratio, and the activated partial thromboplastin time (APTT) [9]. Fibrinolytic coagulopathy, which is independent of consumption coagulopathy but often overlaps with this coagulopathy observed in the analysis, indicates the existence of pathological systemic hyperfibrin(ogen)olysis. Therefore, DIC with the fibrinolytic phenotype, namely coexistence of DIC and systemic hyperfibrin(ogen)olysis, could affect the outcome of trauma patients [2,9]. This review will provide a summary of the recent advances in our understanding of thrombosis and hemostasis following trauma and will discuss the pathogenesis of DIC, especially DIC with the fibrinolytic phenotype at an early stage of trauma.

## Innate immunity, inflammation, and coagulation at the site of injury

Both infectious (sepsis) and non-infectious insults (trauma) can produce a systemic inflammatory response syndrome (SIRS), characterized by systemic proinflammatory cytokine release and generalized activation of leukocytes and the endothelium, leading to multiple organ dysfunction syndrome (MODS). Recent advances in immunology led to the recognition of common ligands involved in SIRS and the understanding that the processes of SIRS are involved in innate immunity [10]. Pathogen-associated molecular patterns (PAMPs) are derived from microorganisms, whereas damage-associated molecular patterns (DAMPs) are molecules produced in stressed or damaged tissues in connection with trauma, shock, ischemia, and reperfusion [10]. The innate immune responses start following the sensing PAMPs or DAMPs by pattern-recognition receptors, such as Toll-like receptors expressed on the immunocompetent cells and endothelium. The sensed danger signals activate both intracellular signal transduction pathways and plasma cascades, which together produce pro-inflammatory cytokines, further stimulating the production of inflammatory biomarkers.

Close interactions between innate immunity, inflammation, and coagulation have been well recognized [11,12]. Innate immune cells have evolved cell-specific prothrombotic pathways that are activated after insults and operate in intact blood vessels to protect hosts from non-self (PAMPs) and altered-self (DAMPs). These processes are called immunothrombosis, and the basic principles have been reviewed [3]. During the responses to PAMPs and DAMPs, monocytes and their microparticles express tissue factor, which activates the extrinsic coagulation pathway [13,14]. Neutrophils are recruited to both infectious and non-infectious sites of inflammation and are activated [15]. When activated, neutrophils release neutrophil extracellular traps (NETs), which are composed of a matrix of DNA, histones, nucleosomes, and antibacterial machinery, including neutrophil elastase, which promotes thrombosis [16]. Histones induce platelet activation and cause profound thrombocytopenia [17]. Histones also promote thrombin generation both by the recruitment of platelets and by impairing thrombomodulin-dependent protein C activation [18,19]. NETs can also activate the intrinsic coagulation pathway by activating FXII to form FXIIa [20], which then promotes the activation of complement pathways. The generated C3a and C5a also promote thrombosis and platelet activation [21]. In addition, extracellular RNA derived from damaged cells constitutes a procoagulant cofactor for the activation of the FXII/FXI-induced coagulation pathway [22]. The neutrophil elastase present on NETs induces the degradation and inactivation of tissue factor pathway inhibitor (TFPI) [23,24] and the thrombomodulin expressed on the endothelium [25,26].

**Figure 1:**
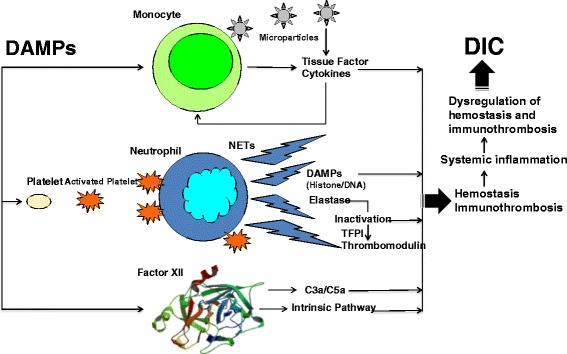
**The pathophysiological processes of local hemostasis, immunothrombosis, and systemic disseminated intravascular coagulation (DIC).** Tissue injury promotes local hemostasis and wound healing. Tissue injury also induces microvascular fibrin thrombosis called immunothrombosis to protect the host from altered-self (damage-associated molecular patterns; DAMPs) and to restrict the DAMPs in the injured vascular compartment. DIC results when local hemostasis and immunothrombosis are no longer able to anchor thrombin or to restrict the spread DAMPs at the injured site. NET, neutrophil extracellular trap; TFPI, tissue factor pathway inhibitor.

All of these changes, including the activation of coagulation and insufficient control of coagulation, give rise to thrombosis at the sites of PAMP- and DAMP-induced inflammation. This local thrombus formation in microvessels impedes the dissemination and tissue invasion of PAMPs and DAMPs as well as pathogens and damaged cells themselves [3,24]. Severe trauma and sepsis are both associated with DIC, a condition that promotes the activation of coagulation and impairs anticoagulation pathways in the systemic circulation [2,27]. In trauma, the thrombin that escapes into the circulation from the injured sites is controlled by antithrombin, TFPI, and thrombomodulin present in intact endothelial cells, as depicted in the cell-based model of hemostasis [28]. However, when systemic inflammation caused by both extensive injuries and shock overwhelms these control mechanisms that restrict the hemostasis locally, DIC ensues [28]. In the same manner, DIC results when immunothrombosis is no longer able to restrict the spread of pathogens or damaged cells at the inflamed injured sites [3,28]. These processes are presented in Figure 1.

## Damage-associated molecular patterns hamper the control mechanisms regulating coagulation and activate coagulation following trauma

The major DAMPs released into the extracellular environment following tissue injury are histones, mitochondrial DNA, nucleosomes, and high-mobility group box 1 (HMGB1) [29]. Within 30 minutes after trauma or immediately after arrival to the emergency department, elevations of the histones and HMGB1 levels have been demonstrated in severely injured trauma patients [30-32]. Thousands of fold-higher levels of mitochondrial DNA have also been observed at a median of 93 minutes after trauma compared with the levels in healthy volunteers [33]. In addition to histones, the HMGB1 released by damaged and inflammatory cells at the site of injury promotes the development of microvascular thrombosis [34]. An important point is that HMGB1 inhibits the anticoagulant protein C pathway mediated by the thrombin-thrombomodulin complex and stimulates tissue factor expression on monocytes. Histones also reduce the cofactor activity of both soluble and endothelial thrombomodulin and impair protein C activation, leading to the subsequent stimulation of plasma thrombin generation [19]. Abrams and colleagues [32] observed significant increases in soluble thrombomodulin and the thrombin antithrombin complex (TAT) associated with IL-6 elevation immediately after histone infusion in a mouse model. The same changes were demonstrated in severely injured trauma patients in the study. In addition, the circulating histone levels were significantly correlated with both the soluble thrombomodulin and TAT levels in trauma patients.

TNF-α and IL-1β are increased immediately after trauma and remain elevated for several days, especially in those with complicated DIC [5,35]. It was found that TNF-α and IL-6 are elevated immediately after histone infusion [32,36]. The early release of IL-6 suggests that it was most likely released from presynthesized stores [32]. TNF-α and IL-1 have been shown to elicit tissue factor formation and expression on the surface of monocytes and endothelial cells. These inflammatory cytokines have been shown to subsequently block the protein C anticoagulant pathway by downregulating thrombomodulin and the endothelial protein C receptor (EPCR) on the endothelium [37]. Furthermore, these inflammatory cytokines activate neutrophils and endothelial cells, leading to neutrophil elastase release from activated neutrophils, which can cleave thrombomodulin, releasing the soluble thrombomodulin from the endothelium in a less active form [25,26,37,38].

These lines of evidence clearly suggest that both DAMPs released from injured cells and tissues and DAMP-induced inflammatory cytokines synergistically hamper the mechanisms controlling coagulation by protein C pathways and activate coagulation, leading to systemic thrombin generation, namely DIC, immediately after trauma.

## Disseminated intravascular coagulation in trauma and traumatic shock

### Definition

The SSC on DIC of the ISTH defined DIC as follows: DIC is an acquired syndrome characterized by the intravascular activation of coagulation with loss of localization arising from different causes. It can originate from and cause damage to the microvasculature, which, if sufficiently severe, can produce organ dysfunction [39]. The most important points of the definition of DIC are ‘intravascular activation of coagulation with loss of localization’ and ‘damage to the microvasculature’, which means thrombin generation and its activation in the circulation and extensive damage to the microvascular endothelium that give rise to insufficient coagulation control.

### Phenotypes

**Figure 2:**
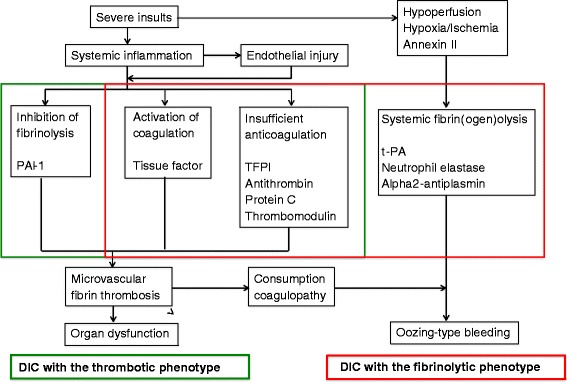
**The two phenotypes of disseminated intravascular coagulation (DIC).** Although the activation of the tissue factor-dependent pathway as the initial step of the coagulation cascade and the presence of insufficient anticoagulation systems are the same, DIC can be subdivided into the fibrinolytic (hemorrhagic) and antifibrinolytic (thrombotic) phenotypes. In DIC with the fibrinolytic phenotype, DIC and systemic fibrin(ogen)olysis coexist. Annexin II expression on the promyelocytes increases the tissue-type plasminogen activator (t-PA) activity in patients with acute promyelocytic leukemia. PAI-1, plasminogen activator inhibitor-1; TFPI, tissue factor pathway inhibitor.

DIC can be subdivided into fibrinolytic (hemorrhagic) and antifibrinolytic (thrombotic) phenotypes [2,5,7] (Figure 2). The sepsis-induced DIC is the thrombotic phenotype [27]. DIC at the early phase of trauma shows the fibrinolytic phenotype, which contributes to massive bleeding and is associated with a poor prognosis [3,7,40]. DIC at the late phase of trauma has a thrombotic phenotype that also affects the patient prognosis by leading to the development of MODS [5,7,27]. The synergistic activation of primary and secondary fibrin(ogen)olysis by tissue-type plasminogen activator (t-PA) is considered to be the cause of DIC with the fibrinolytic phenotype [2], whereas plasminogen activator inhibitor-1 (PAI-1)-mediated inhibition of fibrinolysis causes DIC with the thrombotic phenotype [2,5,7,27]. Activation of coagulation and depression of the inhibitory systems of coagulation are common to both phenotypes.

### Diagnosis

The ISTH and the Japanese Association for Acute Medicine (JAAM) DIC diagnostic criteria have been prospectively validated in patients with critical illness, including trauma [39,41,42]. The JAAM scoring system has validity for diagnosing DIC at an early phase of trauma and also can diagnose DIC with higher sensitivity than the ISTH scoring system. Furthermore, using the JAAM DIC score on admission to the emergency department can independently predict patient death and massive transfusion in trauma cases [40,43,44].

### Impairment of anticoagulation pathways

#### Tissue factor pathway inhibitor

The highly activated tissue factor-dependent pathway is not sufficiently prevented by normal TFPI levels in DIC patients after trauma, because neutrophil elastase cleaves TFPI within the polypeptide that links the first and second Kunitz domains [45]. This impairs the ability of TFPI to neutralize both FXa and the tissue factor/FVIIa complex. The finding suggests that tissue factor and tissue factor/FVIIa complex are continuously formed at a rate that normal TFPI inhibition cannot match [46]. Low levels of antithrombin, protein C activity, and antigens have also been repeatedly confirmed to be present from the early to the late phases of DIC after trauma [7,40,44,47].

#### Thrombomodulin

Higher levels of neutrophil elastase and soluble thrombomodulin have also been confirmed in patients with DIC [47,48]. Soluble thrombomodulin can be formed by the limited proteolysis of endothelial cell membrane thrombomodulin by neutrophil elastase without any evidence of active secretion [25,26]. The amount of soluble thrombomodulin correlates with the degree of endothelial injury [26]. Furthermore, early elevation of TNF-α and IL-1β in DIC patients after trauma causes thrombomodulin downregulation in the endothelium [25,26,35]. Traumatic shock-induced hypoxia leads to a reduction in thrombomodulin and the suppression of thrombomodulin RNA in the endothelium [49,50]. Therefore, the high soluble thrombomodulin levels in patients with DIC suggest a loss of functional thrombomodulin in the endothelium. Furthermore, soluble thrombomodulin has only 20% of the activity of normal endothelial thrombomodulin [51].

#### Protein S and protein C

The thrombin-thrombomodulin complex activates protein C to generate activated protein C. For activated protein C to function, it must form a complex with both protein S and EPCR. The anticoagulant activity of protein S is neutralized by the formation of a complex with complement C4b-binding protein (C4bBP). Increased levels of C4bBP as a consequence of the acute-phase reaction following inflammatory insults result in a relative protein S deficiency, which contributes to the procoagulant state and lethal DIC [52]. Lower levels of protein S activity associated with thrombin generation (prothrombin fragment 1 + 2 (PF1 + 2)) have already been demonstrated in trauma patients immediately after arrival to the emergency department [53].

Activated protein C is immediately inactivated by protease inhibitors, such as the protein C inhibitors, α1-antitrypsin, α2-antiplasmin, and α2-macroglobulin. In cases of DIC due to trauma, lower protein C and protein S levels, relative protein S deficiency, and impaired functions of both soluble and endothelial thrombomodulin are all implicated in the insufficient conversion of protein C to activated protein C and the inability of activated protein C. Therefore, increases in activated protein C levels do not indicate a shutoff of thrombin generation. This fact was confirmed in patients with severe sepsis, and the downregulation of endothelial thrombomodulin and the dysfunction of the endothelium were assumed to be the mechanisms responsible for this finding [54]. In the reported study, the dissociation between PF1 + 2 and the activated protein C levels was observed in 66% of patients. In fact, the elevated activated protein C levels (~10 ng/mL) did not reach a concentration (70 to 80 ng/mL) sufficient to inhibit thrombin generation in severely injured trauma patients associated with tissue hypoperfusion [54,55].

#### Antithrombin

Antithrombin inactivates thrombin and inhibits several proteases of both the extrinsic and intrinsic coagulation pathways, including FIXa, FXa, FXIa, and FXIIa. Therefore, reductions of antithrombin can significantly influence the coagulation processes, and are a potential risk factor for thrombosis [56]. Insufficient levels of antithrombin compared with the potential for thrombin generation in the prothrombin complex concentrate induced DIC in a pig model of coagulopathy with blunt liver injury [57]. The severity of injury and tissue hypoperfusion are major contributors to the reduction of antithrombin in trauma [58,59]. Low antithrombin levels are associated with thromboembolic complications, and the process continues to DIC [60]. Extremely low levels of antithrombin could be observed in cases of trauma with DIC immediately after arrival to the emergency department [44,61]. This reduction of antithrombin persisted several days after admission [44,61,62].

Two studies showed that a decreased ability to localize hemostasis at the wound site and subsequent systemic thrombin generation are the results of decreased antithrombin levels in patients with coagulopathy immediately after trauma [63,64]. Similarly, a multiple regression analysis demonstrated that the antithrombin levels are an independent determinant of the high levels of soluble fibrin, a marker of thrombin generation and activity, in DIC patients after trauma [62]. Furthermore, a rat model of DIC by Noble-Collip drum trauma and shock confirmed that the antithrombin levels negatively correlate with thrombin generation (Gando, unpublished data).

**Figure 3:**
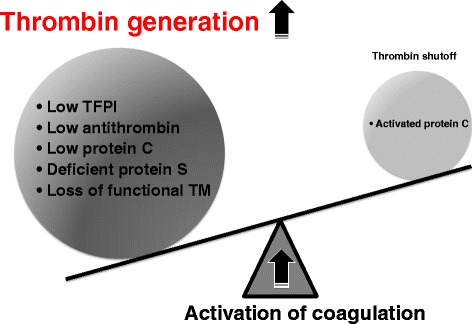
**The balance between thrombin generation and its inhibition.** Disseminated intravascular coagulation occurs when there is an imbalance between thrombin generation and its inhibition. Insufficient coagulation control mechanisms contribute to massive thrombin generation in the circulation, which overwhelms activated protein C-mediated inhibition of thrombin generation. TFPI, tissue factor pathway inhibitor; TM, thrombomodulin.

These results clearly indicate that there is a much lower availability of the TFPI, antithrombin/glycosaminoglycan, and thrombomodulin/protein C systems for the regulation of thrombin generation and its activation in patients with DIC. Furthermore, higher soluble thrombomodulin levels suggest the presence of extensive damage to microvasculature endothelium. These changes are summarized in Figure 3.

### Thrombin generation in the systemic circulation

Thrombin generation is essential to control the four major hemostatic domains, consisting of coagulation, anticoagulation, fibrinolysis, and antifibrinolysis, and the endothelial function deeply affects all four of these hemostatic systems [65]. Under normal conditions, thrombin generation and clot formation are localized to the site of the injured vessels associated with these systems, as shown in the cell-based model [28,65]. However, systemic inflammation caused by severe insults can alter the properties of the four hemostatic systems by affecting the endothelial function. Systemic thrombosis, namely DIC, ensues when these changes overwhelm the anticoagulation mechanisms that restrict the hemostasis locally [28,65].

Soluble fibrin and fibrinopeptide A are considered to be accurate markers of thrombin generation and activity because both of these are formed as a result of the direct action of thrombin on fibrinogen, which is followed by fibrin formation. Extremely elevated levels of fibrinopeptide A in trauma patients with DIC immediately after the arrival to the emergency department have been described [61]. In addition, higher levels of these molecular markers of thrombin generation at an early stage of trauma have been repeatedly confirmed [5-7,47,48,53]. Dunbar and Chandler demonstrated excessive non-wound-related thrombin generation in trauma patients with both DIC and ‘ACOTS’ immediately after arrival to the hospital [63,64]. Their first study showed marked systemic thrombin generation due to circulating procoagulants that initiate thrombin generation systemically as well as a reduced ability to localize hemostasis at the wound site due to the loss of antithrombin. Their second study found that tissue factor activity made up approximately 80% of the total procoagulant activity. Reports showing a significant correlation between tissue factor and the markers of thrombin generation and microparticle formation by activated platelets support these results [66,67]. Importantly, the term ‘ACOTS’ in the first study was changed to ‘DIC’ in their second study [63,64].

The overall function of the thrombomodulin/protein C anticoagulant pathway can be precisely evaluated by examining the prothrombinase activity [68]. Prothrombinase is a complex comprising FXa, FVa, phospholipids, and Ca^2+^ and is the biggest determinant of thrombin generation from prothrombin. The prothrombinase activity, measured as the thrombin generation rate, decreases in proportion to the amount of thrombin-thrombomodulin complex-induced formation of activated protein C and the subsequent inactivation of FVa [68,69]. DIC patients after trauma showed normal prothrombinase activity associated with higher levels of soluble fibrin [62]. These results suggest that the inhibition of the prothrombinase activity caused by activated protein C-mediated anticoagulation does not overwhelm the activation of the tissue factor-induced systemic thrombin generation or its activation in traumatic DIC patients. This imbalance between thrombin generation (soluble fibrin) and its inhibition (prothrombinase activity) could be explained by an insufficiency in the other anticoagulant mechanisms, such as TFPI and antithrombin, and a lower function of thrombomodulin due to endothelial injury, which were confirmed in that study [62]. Importantly, all of the above-mentioned results were also observed in patients diagnosed with ACOTS [62].

### Consumption coagulopathy

DIC has been recognized as a consumptive thrombohemorrhagic disorder [2]. Consumptive processes reflect the multiple actions of thrombin. Increased thrombin generation accounts for decreases in platelets, fibrinogen, FII, FV, FVIII, and FXIII in acute consumption, and the decreases in other clotting factors, such as FIX and FX, are due to the rapid clearance of activated clotting factors *in vivo* [2]. Thrombin induces the release of t-PA from endothelial cells, followed by plasmin generation. If plasmin is formed sufficiently in the circulation, it degrades fibrinogen, FV, and FVIII. These lines of evidence support a rapid consumption of thrombin-sensitive hemostatic factors, including platelets, fibrinogen, and factors V, VIII, and XIII. As a result of thrombin-mediated protein C activation, sensitive and rapid decreases in the levels of FV and FVIII have been demonstrated in pre-DIC and DIC cases [70,71]. In cases of DIC due to trauma, platelets are sometimes consumed slowly because of marginalization in blood vessels and the release from storage in organs such as the spleen, liver, and lungs [5,7,72]. FVIII is known to paradoxically increase in response to clinical insults, including trauma, due to release of von Willebrand factor (VWF) from the endothelial Weibel-Palade bodies [73] and the acute phase behavior of FVIII. The VWF immediately interacts with FVIII, and this interaction prolongs the plasma half-life of FVIII [74]. The consumption of coagulation factors prolongs both the prothrombin time (PT) and APTT; however, the APTT sometimes is normal or even shortened because of interactions between FVIII with VWF in spite of a prolongation of the PT in patients with DIC.

A prolonged PT, which reflects a decrease in FV and to a lesser extent in factors II, VII, and X, and decreases in fibrinogen levels immediately to several days after trauma have been repeatedly confirmed in cases of trauma with DIC [5,7,35,40,43,47,61,62,66]. A prolonged APTT, which reflects a decrease in factors V, VIII, and fibrinogen, has also been confirmed immediately after trauma in patients with DIC [61]. FVII antigen has been demonstrated to be consumed at a relatively slow speed for about 8 hours in a rabbit model of DIC [75]. Importantly, the FVIIa levels increased to 120% within 2 hours after the induction of DIC and then declined. Furthermore, the FXIII, α2-antiplasmin and fibronectin levels, which play important roles in cross-linking fibrin formation and wound healing, showed marked and rapid decreases in DIC patients at arrival to the emergency department [76].

The consumption of coagulation factors, especially FV and FVIII, is a basic principle of DIC in trauma, which was confirmed several decades ago [77]. A recent principle component analysis reconfirmed that global coagulation factor consumption, as well as decreased protein C and antithrombin levels, is associated with an increased incidence of coagulopathy and the mortality of the patients [9].

### Activation of fibrinolysis

DIC and pathological systemic fibrin(ogen)olysis sometimes coexist following the same insult, including trauma, which is called DIC with the fibrinolytic phenotype [2]. Traumatic shock-induced tissue hypoperfusion causes t-PA release from the endothelial Weibel-Palade bodies, which leads to systemic fibrin(ogen)olysis in addition to DIC-induced secondary fibrinolysis [2,73]. Increased fibrinolysis, as well as the activation of coagulation in trauma, has long been recognized [78,79]. Recently, these findings were reconfirmed in severely injured trauma patients, 40% of whom showed a PT ratio of more than 1.2 [80]. That study demonstrated increased thrombin generation and consumption of fibrinogen and antithrombin, as well as increased t-PA, plasmin generation, and fibrinolysis along with consumption of α2-antiplasmin, all of which coincided with DIC with the fibrinolytic phenotype.

The most important point in the pathogenesis of fibrin(ogen)olysis at an early stage of trauma is that there is a several hour time difference between the immediate t-PA release from the endothelium and later expression of PAI-1 mRNA, leading to an extreme imbalance of these molecules [81-83]. Supporting this imbalance, the levels of PAI-1 are almost identical in patients with and without DIC immediately after trauma, whereas the levels of t-PA and plasmin generation both were significantly increased in patients with DIC [5,35,61,62].

In addition to plasmin, neutrophil elastase-mediated fibrinolysis is involved in the pathogenesis of fibrin(ogen)olysis in DIC with the fibrinolytic phenotype [47]. The lower levels of α2-antiplasmin, FXIII, and fibronectin in patients with DIC suggest that there is insufficient inhibition of plasmin, impaired cross-linking of fibrin, and delayed wound healing, leading to fragile fibrin formation associated with persistent bleeding [5,7,61,76]. A study showing tissue factor-induced fibrin(ogen)olysis without tissue hypoperfusion suggests that secondary fibrinolysis caused by massive fibrin formation-induced t-PA release may also have a role in DIC with the fibrinolytic phenotype [84]. Importantly, thrombomodulin-mediated thrombin-activatable fibrinolysis inhibitor activation does not have an important role in the pathogeneses of fibrin(ogen)olysis immediately after trauma, which indirectly supports that there is insufficient thrombomodulin/protein C pathway. Therefore, activated protein C-mediated inhibition of PAI-1 is unlikely [47,80].

## Solving the mystery of microvascular thrombosis

**Figure 4:**
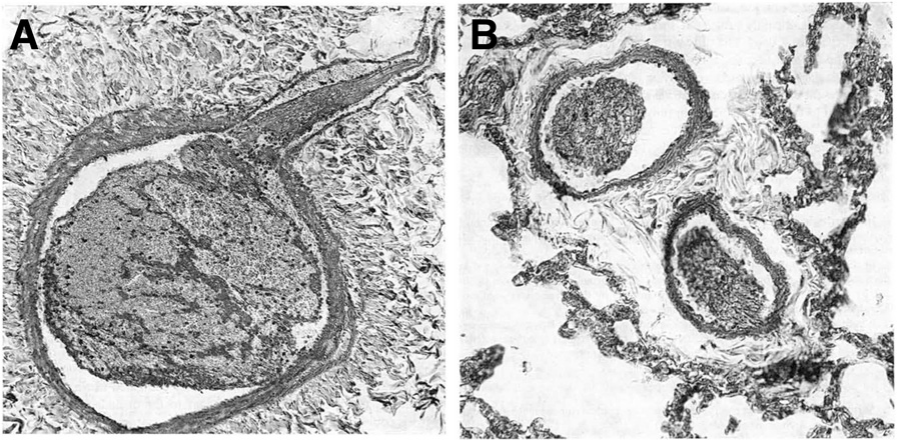
**The inhibition of the activation of fibrinolysis by tranexamic acid revealed microvascular thrombosis and thromboemboli formation in the large vessels in a dog model of hemorrhagic shock. (A)** A section of a branch of the portal vein almost completely filled by a mixed thrombus consisting of platelets and fibrin threads in bundles. **(B)** A section from lung vessels with thrombotic masses consisting of both platelets and fibrin thread. Phosphotungstic acid hematoxylin staining. Adapted with permission from Leandoer and Bergentz [91].

Histological evidence of microvascular thrombosis in DIC, especially in DIC with the thrombotic phenotype, has been reported in clinical, experimental, and autopsy findings [85]. However, this evidence of DIC with the fibrinolytic phenotype is rarely available in humans and was extensively debated during the 1960s and 1970s [86]. These debates on the inconsistency of thrombus formation had come about due to the existence of hyperfibrin(ogen)olysis at an early stage of trauma and traumatic shock. Although arguments had been proposed, fibrin thrombosis [87], vein thrombi formation [88], platelet aggregation, and emboli formation [89,90] had been repeatedly confirmed in hemorrhagic shock and trauma. Subsequently, the platelet and fibrin thrombosis became more prominent during antifibrinolytic therapy using tranexamic acid in a dog model of hemorrhagic shock [91] (Figure 4). It should be emphasized that the article expressing negative opinions about DIC had been forced to conclude that some fibrin thrombi were observed in their histological study [92]. Importantly, signs of inflammation, microthrombus, and embolus formation could be observed within 24 hours after trauma in human studies [93,94]. As it is clearly understood according to the definition of DIC proposed by the SSC on DIC of ISTH, it is of limited interest to discuss whether microvascular thrombosis occurs in a situation of fibrin(ogen)olysis immediately after trauma and traumatic shock. However, it is important to recognize the coexistence of pro- and anti-thrombotic states during the early stage of trauma. Therefore, the debate on microvascular thrombosis has major implications for the therapeutic strategies for patients with DIC after trauma [95].

## Animal models

Animal models of trauma have their limitations, and the findings in animals may not directly mimic the situation in trauma patients. Two review articles identified a general lack of experimental research to keep pace with the research on human trauma [96,97]. Differences in the coagulation systems between different species are also an important issue. Porcine models are relatively hypercoagulable and have only 36% of the levels of protein C [97]. Many different kinds of trauma models have been used in experimental animal studies, which have resulted in diverse interpretations of trauma-induced coagulopathy [96,97]. However, under these circumstances, the superiority of the polytrauma and shock model has recently been reported [98].

The Noble-Collip drum-induced polytrauma and traumatic shock uncomplicated by gross hemorrhage have been used by numerous investigators to mimic lethal traumatic injury. Noble-Collip drum trauma and shock could immediately reproduce typical DIC with the fibrinolytic phenotype, with animals exhibiting decreases in platelet counts, the prolongation of the PT and APTT, decreases in fibrinogen and antithrombin, and elevation of the fibrin/fibrinogen degradation products [99,100]. Furthermore, the elevation of t-PA, shortening the euglobulin lysis time, and decrease in α2-antiplasmin indicated the immediate activation of the fibrinolytic system [100]. Decreases in the levels of FXII, prekallikrein, and CH50 suggested the activation of both the intrinsic coagulation pathway and the complement system [100]. Immediately after Noble-Collip drum trauma and shock, tissue factor increases in the circulation and its mRNA expression has been observed in various organs, indicating the activation of the extrinsic coagulation pathway [101].

Tissue factor-induced DIC model has been reported to be a ‘grade A’ relevant experimental model for trauma [96]. This model demonstrated that a massive amount of tissue factor induces DIC associated with fibrin(ogen)olysis without tissue hypoperfusion [84]. This result suggests that trauma itself could give rise to DIC without tissue hypoperfusion, thus supporting the results of a previous clinical study [47]. Tissue factor-induced activation of coagulation leads to generalized consumption of not only coagulation factors but also of the inhibitory feedback factors involved in controlling coagulation and fibrinolysis, namely antithrombin and α2 antiplasmin, respectively. Lower levels of antithrombin enhance thrombin generation, and a decrease in the level of α2 antiplasmin is another important factor involved in the pathogenesis of hyperfibrin(ogen)olysis, which is in agreement with the results of clinical studies [63,64,76].

## Conclusions

The main pathophysiological mechanism underlying the hemostatic changes that occur following trauma and traumatic shock is DIC with the fibrinolytic phenotype. This is associated with activation of coagulation, insufficient control of coagulation, fibrin(ogen)olysis, and consumption coagulopathy, leading to oozing-type bleeding at mucosal lesions, serosal surfaces, and surgical-site wounds, which affect the prognosis of trauma patients. The pathogenesis of DIC with the fibrinolytic phenotype is due to the dysregulation of local hemostasis and immunothrombosis that are overwhelmed by the systemic inflammation caused by extensive tissue injury and tissue hypoperfusion. Traumatic shock-induced hypoperfusion and fibrin thrombi give rise to primary and secondary fibrin(ogen)olysis due to the t-PA released from the endothelium. The coexistence of hypothermia, acidosis, and dilution aggravates DIC with the fibrinolytic phenotype and then leads to so-called trauma-induced coagulopathy.

## Abbreviations

ACOTS: Acute coagulopathy of trauma shock
APTT: Activated partial thromboplastin time
C4bBP: C4b-binding protein
DAMP: Damage-associated molecular pattern
DIC: Disseminated intravascular coagulation
EPCR: Endothelial protein C receptor
HMGB1: High-mobility group box 1
IL: Interleukin
ISTH: International Society on Thrombosis and Haemostasis
JAAM: Japanese Association for Acute Medicine
MODS: Multiple organ dysfunction syndrome
NET: Neutrophil extracellular trap
PAI-1: Plasminogen activator inhibitor-1
PAMP: Pathogen-associated molecular pattern
PF1 + 2: Prothrombin fragment 1 + 2
PT: Prothrombin time
SIRS: Systemic inflammatory response syndrome
SSC: Scientific and Standardization Committee
TAT: Thrombin antithrombin complex
TFPI: Tissue factor pathway inhibitor
TNF-α: Tumor necrosis factor α
t-PA: Tissue-type plasminogen activator
VWF: von Willebrand factor
